# Prevalence of Methicillin-resistant *Staphylococcus Aureus* (MRSA) in a Tertiary Care Hospital in Northern India

**DOI:** 10.4103/0974-2727.72154

**Published:** 2010

**Authors:** Shilpa Arora, Pushpa Devi, Usha Arora, Bimla Devi

**Affiliations:** Department of Microbiology, Govt. Medical College, Amritsar, India

**Keywords:** Glycopeptides, MRSA, multidrug resistance

## Abstract

**Aim:**

The emergence of *Methicillin-resistant Staphylococcus aureus* (MRSA) has posed a serious therapeutic challenge. We report the prevalence and antibiotic susceptibility pattern of MRSA in the hospitals attached to GMC, Amritsar, Punjab.

**Materials and Methods:**

The study comprised of 250 coagulase-positive staphylococci (COPS) isolated from a total of 6743 clinical specimens (like pus, blood, urine, high vaginal swab, sputum, etc.) of patients admitted in hospitals attached to Government Medical College, Amritsar from January 2008–February 2009. Routine antibiotic susceptibility testing was performed and interpreted as per standard guidelines. Methicillin resistance was detected using oxacillin and cefoxitin disc diffusion method, oxacillin screen agar method, and minimum inhibitory concentration using broth macrodilution method.

**Results:**

A total of 115 (46%) strains were found to be methicillin resistant. Multidrug resistance was observed in 73% MRSA strains. However, no strain was resistant to vancomycin.

**Conclusion:**

Regular surveillance of hospital-associated infection and monitoring of antibiotic sensitivity pattern is required to reduce MRSA prevalence.

## INTRODUCTION

*Staphylococcus aureus* is responsible for causing a variety of human infections, which may range from minor skin diseases to life-threatening infections.[1] It colonizes healthy individuals and causes severe infection in hospitalized patients. *Staphylococcus aureus* infections used to respond to ß-lactam and related group of antibiotics but the emergence of *Methicillin-resistant S. aureus* (MRSA) has posed a serious therapeutic challenge.[2]

Infected and colonized patients in hospitals mediate the dissemination of MRSA strains, and hospital staff is the main source of transmission. This leads to serious endemic and epidemic MRSA infections.[3] The possible predisposing factors that increase the chance of emergence and spread of MRSA are prolonged and repeated hospitalization, indiscriminate use of antibiotics, lack of awareness, intravenous drug abuse, and presence of indwelling medical devices.[4]

MRSA strains are difficult to eradicate as they are multidrug-resistant leaving glycopeptides as the drugs of choice.[1] Resistance has been reported to these drugs also from various parts of the country.[5,6] The knowledge of prevalence of MRSA and their antimicrobial-susceptibility pattern is a must for appropriate treatment of these infections. The present study was conducted to know the prevalence of MRSA in our hospital, which is a tertiary referral hospital.

## MATERIALS AND METHODS

The study comprised of 250 coagulase-positive staphylococci (COPS), isolated from a total of 6743 clinical specimens (like pus, blood, urine, high vaginal swab, sputum, etc.) of patients admitted in hospitals attached to GMC, Amritsar. All the isolates were identified by standard procedures[7] (gram staining, catalase test, mannitol fermentation, slide coagulase and tube coagulase test). Tube coagulase was taken as the main criteria of identification and was performed by diluting rabbit plasma in freshly prepared normal saline (1:6). Three to four colonies were emulsified in 1 ml of diluted plasma and the tubes were incubated at 37°C. Readings were taken at 1, 2, 3 and 4 h and further incubated at room temperature if no clot formation was observed. Out of 250 tube coagulase-positive strains, slide coagulase was positive in 233 strains.

All the strains were then subjected to antimicrobial-susceptibility testing by Kirby–Bauer disc diffusion method. Antibiotics tested were penicillin (10 units), oxacillin (1 μg), cefoxitin (30 μg), erythromycin (15 μg), cephalexin (30 μg), ciprofloxacin (5 μg), gentamicin (10 μg), amikacin (30 μg), linezolid (30 μg), vancomycin (30 μg), norfloxacin (10 μg) and nitrofurantoin (300 μg) (Hi Media Mumbai). Norfloxacin and nitrofurantoin were used only in urine samples, while erythromycin was not put up in these samples. Oxacillin disc was put on a separate Mueller Hinton Agar (MHA) (Hi Media, Mumbai) plate supplemented with 4% NaCl. Zone diameters were measured following CLSI criteria.[8] Transmitted light was used to examine the oxacillin and vancomycin zones. ATCC 29213 strain was used as a reference strain.

All the isolates were tested for methicillin resistance by oxacillin screen agar (OSA) method and broth macrodilution method (for knowing minimum inhibitory concentration (MIC)) as well.[9]

Oxacillin screen agar – Using a swab, the 0.5 McFarland suspension of the isolate was spotted on the MHA plate containing 6 μg/ml oxacillin and 4% NaCl in 10–15 mm area. Plates were observed carefully in transmitted light. Any visible growth after 24 h of incubation at 35°C was indicative of resistance.

Oxacillin MIC – Serial dilutions ranging from 0.25 to 256 μg/ml of oxacillin were prepared in Mueller Hinton Broth (MHB) (Hi Media Mumbai) containing 2% NaCl. The inoculum was prepared by diluting 0.5 McFarland suspension to the concentration of 10^5^ CFU/ml. The tubes were inoculated and incubated at 35°C for 24 h. The lowest concentration at which there was no visible growth was taken as the MIC. The strains for which MIC was > 2 μg/ml were considered resistant.

## RESULTS

The distribution pattern of 250 *S. aureus* strains isolated from various specimens and wards is shown in Table 1. Antibiotic sensitivity pattern of all the isolates, and comparison between MRSA and MSSA strains is shown in Table 2 and 3.

**Table 1:** Distribution of various samples from different wards

	Pus	Urine	Vaginal swab	Blood	Drain tip	Sputum	Total
Orthopedics	74	-	-	1	-	-	75
Pediatrics	2	2	-	52	-	-	56
Medicine	7	8	-	23	3	3	44
Gynecology	5	15	6	1	-	-	27
Surgery	24	2	-	-	-	-	26
Skin STD	7	-	-	-	-	-	7
Chest TB	2	-	-	-	2	2	6
ENT	5	-	-	-	-	-	5
ART	-	-	-	2	-	-	2
GDC	1	-	-	-	-	-	1
Plastic surgery	1	-	-	-	-	-	1
Total	128	27	6	79	5	5	250

GDC= Govt. Dental college

**Table 2:** Antibiogram of Staphylococcus aureus strains

Name of antibiotic	No. of strains	Sensitive		Intemediate		Resistant	
		No	Percentage	No	Percentage	No	Percentage
Pencillin	250	54	21.6	-	-	196	78.4
Oxacillin	250	145	58.0	4	1.6	101	40.4
Cefoxitin	250	135	54.0	-	-	115	46.0
Erythromycin^a^	223	111	49.8	-	-	112	50.2
Cephalexin	250	108	43.2	-	-	142	56.8
Ciprofloxacin	250	118	47.2	-	-	132	52.8
Gentamicin	250	115	46.0	-	-	135	54.0
Amikacin	250	195	78.0	-	-	55	22.0
Nitrofurantoin□	27	12	44.4	-	-	15	55.6
Norfloxacin□	27	12	44.4	-	-	15	55.6
Linezolid	250	248	99.2	-	-	2	00.8
Vancomycin	250	250	100.0	-	-	0	00.0

a not put in urine samples; □put up only in urine samples

**Table 3:** Resistance of MRSA and MSSA strains to individual antimicrobial agents

Antibiotic	MSSA			MRSA		
	No. of strains	Resistant	%	No. of strains	Resistant	%
Pencillin	135	81	60.0	115	115	100.0
Erythromycin°	121	49	40.5	102	63	61.7
Cephalexin	135	49	36.3	115	93	80.9
Ciprofloxacin	135	54	40.0	115	78	67.8
Gentamicin	135	52	38.5	115	83	72.2
Amikacin	135	12	08.9	115	43	37.4
Nitrofurantoin□	14	7	50.0	13	8	61.5
Norfloxacin□	14	8	57.1	13	7	53.8
Linezolid	135	0	00.0	115	2	01.7
Vancomycin	135	0	00.0	115	0	00.0

° not put in urine samples; □put up only in urine samples; MRSA = Methicillinresistant *S. aureus*; MSSA= Methicillin-sensitive *S. aureus*

**Figure 1:**
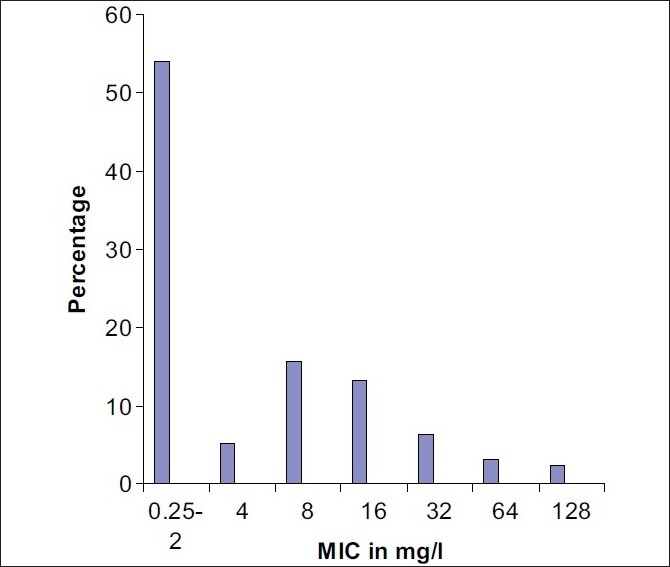
MIC of oxacillin in *Staphylococcus aureus* strains

Out of 250 *S. aureus* isolates, 101 (40.4%) and 4 (1.6%) strains were found to be resistant and intermediate resistant by using oxacillin disc diffusion method. By cefoxitin disc, OSA and MIC testing, methicillin resistance was detected in 115 (46%) strains. MIC of oxacillin in various strains is shown in Figure 1.

Seventy three percent (84/115) MRSA strains were observed to be resistant to ≥3 drugs other than penicillin and were considered as multidrug-resistant (MDR). Two MRSA strains were resistant to six antibiotics, 21 to five, 31 to four, 30 to three, 16 to two and 14 to one antibiotic (excluding penicillin). One MRSA strain was resistant to only penicillin out of the various antibiotics used. All *S. aureus* strains including MRSA were however found to be sensitive to vancomycin.

Orthopedics patients accounted for the maximum number of MRSA strains (32) followed by pediatrics (28), medicine (20), gynecology (12), surgery (11), skin STD (5), chest TB and ART (2 each), and ENT, plastic surgery and dental surgery (1 each).

## DISCUSSION

It is worrisome that the present study reports an alarmingly high prevalence (46%) of MRSA infection. Other studies have also shown such a high MRSA prevalence in various parts of the country ranging from 40.6% to 54.85% to 59.3%.[2,4,6] However, 31.1 and 23.6% MRSA prevalence has also been reported,[3,10] which is comparatively less than that reported in the present study. This variation might be because of several factors like efficacy of infection control practices, healthcare facilities and antibiotic usage that vary from hospital to hospital.

In the present study 63 (54.8%) MRSA strains were isolated from surgical units, and orthopedics ward alone accounted for 32 (27.8%) resistant strains. Srinivasan S *et al*. also found that surgical units accounted for 80% of the MRSA isolates and postoperative infections in orthopedic surgery accounted for 28%.[11]

The difficulty in detection of methicillin resistance is because of its heterogenous nature. Cefoxitin was found to be superior to oxacillin disc for detection of methicillin resistance in the present study as it detected significantly higher number of strains than oxacillin disc (*P*-value < 0.05) taking MIC broth macrodilution method as the standard. Moreover it is easy to put up and interpret. Although OSA method could also detect equivalent number of resistant strains as that detected by cefoxitin disc, it is however cumbersome to perform. The superiority of cefoxitin disc has been reported by other authors as well.[12,13]

The association of multidrug resistance with MRSA adds to the problem and it is rightly said that hospital dust is more dangerous than roadside dust and danger is from MRSA. MRSA strains were found to be more resistant to other antibiotics than MSSA strains. Significant difference (*P*-value < 0.05) was observed in case of penicillin, erythromycin, cephalexin, ciprofloxacin, gentamicin and amikacin. However, the difference observed in case of norfloxacin, nitrofurantoin and linezolid was statistically insignificant (*P*-value > 0.05). Vidhani S *et al*. also found that there was a marked difference between sensitivity pattern of MRSA and MSSA isolates.[14] Majumder D *et al*. also reported that coexisting resistance to different antibiotics (except penicillin) with methicillin was significantly higher in comparison to methicillin-sensitive strains.[10]

All MRSA strains were sensitive to vancomycin in the present study. This is in accordance with other studies.[1,3,4] However, vancomycin-intermediate and vancomycin-resistant *S. aureus* (VISA and VRSA) strains have been reported recently from various parts of the country.[5,6]

The percentage of MDR strains among MRSA was found to be 73%. In the various reports from other parts of the country, the burden of such strains has ranged from 23.2% to 32% to 63.6%.[3,4,10] In a study done in Nepal, MDR MRSA was reported to be 40.1%.[1] Reporting of high rates of MDR MRSA leads to the possibility of exploitation of vancomycin by clinicians. A 100% sensitivity of MRSA to vancomycin suggests its prudent use and continuous monitoring of MIC levels so that we may not fall back into pre-antibiotic era.

To conclude, glycopeptides seems to be the only antimicrobial agents that may be used as the drug of choice to treat MDR MRSA infections. The high prevalence of MRSA and glycopeptide use, both thought to be risk factors for VRSA, make the widespread dissemination of these organisms an alarming and realistic possibility once it happens to emerge. So, glycopeptides must be kept reserved for life-threatening infections caused by MDR MRSA.

The marked difference between antibiogram of MRSA and MSSA isolates calls for the routine testing of methicillin-resistance, which may preferably be done by using cefoxitin disc.

The most effective way to prevent MRSA infections is by doing continuous surveillance of antibiotic resistance profiles of local *S. aureus* isolates to formulate antibiotic policies and effective infection control practices.
